# Combinations of mental disorders and their association with mortality in the UK Biobank

**DOI:** 10.1186/s12888-026-07865-w

**Published:** 2026-02-24

**Authors:** Vivian Boschesi Barros, Alexandre Dias Porto Chiavegatto Filho

**Affiliations:** Department of Epidemiology, School of Public Health, University of São Paulo, Av. Dr. Arnaldo, 715, São Paulo, SP 01246-904 Brazil

**Keywords:** Mortality, Psychiatry, Mental health, Epidemiology

## Abstract

**Objectives:**

To explore patterns of combinations of mental disorders and their association with mortality using data from the UK Biobank, a large middle-aged and elderly cohort.

**Methods:**

This longitudinal cohort study analyzed data from approximately 160,000 UK Biobank participants who completed a Mental Health Questionnaire. We identified six probable lifetime mental disorders, examined their combinations using association rule mining, and estimated age and sex-adjusted mortality rate ratios (MRRs) for each disorder and combination.

**Results:**

Combinations of mental disorders were found in approximately 30% of questionnaire completers identified with at least one probable lifetime mental disorder. Most combinations involved depression and/or anxiety. Combinations of mental disorders were generally associated with higher mortality, especially those including alcohol and substance use disorders. The highest MRR was observed for alcohol use disorder plus substance use disorder plus psychotic experience (MRR = 4.94, 95% CI [2.57–9.5]).

**Conclusions:**

In a large middle-aged and elderly cohort in the UK, combinations of probable lifetime mental disorders identified via an online questionnaire were common and generally associated with higher mortality.

**Clinical trial number:**

Not applicable.

**Supplementary Information:**

The online version contains supplementary material available at 10.1186/s12888-026-07865-w.

## Introduction

Cohort studies and meta-analyses have consistently associated practically all mental disorders with increases in mortality [1–3]. According to a meta-review by Chesney et al. (2014) [1], the all-cause mortality risk estimate associated with specific mental disorders varied from 1.4 in dysthymic disorder to 14.7 in opiate use disorder. However, mental disorders commonly co-occur. Large-scale community surveys reported that more than 50% of people with a lifetime history of mental disorder had at least one additional lifetime mental disorder [4]. Besides being concerningly prevalent, combinations of mental disorders are associated with greater disorder severity and worse prognosis [4–7]. However, very few studies have explored the extent of their impact on mortality [8]. This line of research has clinical and public health relevance, as it can reveal subgroups of people with mental disorders at a particularly high risk of death.

To our knowledge, the first large comprehensive study on the impact of combinations of mental disorders on mortality was published by Plana-Ripoll et al. (2020) [8]. This study included more than seven million people who lived in Denmark at any time between 1995 and 2016 and used data from national inpatient, outpatient, and emergency medical registers. The authors showed that most combinations of mental disorders were associated with increased mortality, especially those including substance use disorders. The combination of mental disorders associated with the highest mortality rate ratio in this study was schizophrenia, neurotic disorders, and substance use disorders (MRR = 6.0). Comparatively, each of these disorders was individually associated with mortality rate ratios of 2.6, 2.1, and 3.9, respectively, in a previous Danish study from the same research group [2]. These findings suggest that combinations of mental disorders might account for an important share of the excess mortality associated with mental disorders.

Registry-based studies, such as the one by Plana-Ripoll et al. (2020) [8], have many strengths. This type of data source can provide access to large samples followed for extended periods and typically allows the identification of a wide range of mental disorders. However, one of the main limitations of registry-based studies is that they usually include a high proportion of severe or inpatient cases. The study by Plana‐Ripoll et al. (2020), for instance, did not include mental disorder cases treated exclusively in primary care. Furthermore, registry-based studies can only identify mental disorder cases recorded by healthcare providers. However, many mild cases of mental disorders never receive medical attention. For these reasons, registry-based studies tend to overestimate the relative mortality associated with mental disorders. This idea is supported by a meta-analysis showing that common mental disorders are associated with a relative risk of death of 2.4 in registry-based studies versus 1.9 in psychiatric interview-based studies (even though this difference was not considered statistically significant) [3].

We are unaware of non-registry-based studies on the mortality associated with combinations of mental disorders. For this reason, we explored this topic using data from the UK Biobank. The UK Biobank is a cohort study that recruited around half a million middle-aged and elderly participants between 2006 and 2010 [9]. These participants underwent a comprehensive baseline assessment of their sociodemographic and health-related history and are having their morbimortality monitored over time. Around 160,000 UK Biobank participants completed an online Mental Health Questionnaire, which was implemented in 2016 [10]. This questionnaire is mainly based on validated tools that identify psychiatric symptoms and can be used to identify probable cases of common mental disorders. This approach captures milder and untreated cases often missed by clinical registers, potentially reducing the overrepresentation of severe cases typical of registry-based studies. In a previous publication, our research group already used data from the UK Biobank Mental Health Questionnaire to investigate the association between common mental disorders and mortality [11].

Based on prior evidence, we hypothesized that combinations of mental disorders would be common among UK Biobank Mental Health Questionnaire completers and associated with higher mortality. To test these hypotheses, our objectives were to use data from the UK Biobank Mental Health Questionnaire to (1) study patterns of combinations of probable lifetime mental disorders using an association rule mining approach and (2) estimate the mortality rate ratios associated with these combinations.

## Materials and methods

### Study design

This is a longitudinal cohort study using data from the UK Biobank and its Mental Health Questionnaire to explore patterns of combinations of mental disorders and their association with mortality. Our study methodology is described below according to the Reporting of Observational Studies in Epidemiology (STROBE) statement [12]. 

### Setting

The UK Biobank is a large cohort study that recruited approximately 500,000 middle-aged and elderly participants from the United Kingdom between 2006 and 2010 [9, 13]. UK Biobank participants underwent a comprehensive baseline assessment and are being followed up long-term [13]. They are also periodically invited to participate in additional assessments, including an online Mental Health Questionnaire [10, 14].

### Data collection

The baseline assessment of UK Biobank participants was performed in 22 assessment centers located across England, Scotland, and Wales [9]. During this baseline assessment, UK Biobank participants provided sociodemographic and health-related information and consented to the periodic collection of information about their health status and mortality via linkage with databases from the UK public National Health System [13].

In 2016, the UK Biobank implemented the Mental Health Questionnaire, developed mostly using validated instruments (such as the World Health Organization Composite International Diagnostic Interview Short-Form (CIDI-SF), modified to provide lifetime history), as described by Davis et al. (2020) [10]. Questionnaire responses can be used in scoring systems to identify probable mental disorders [15]. For brevity, we sometimes use the term “mental disorder” to refer to probable mental disorders identified through the questionnaire. However, these should not be interpreted as confirmed clinical diagnoses.

### Participants

The UK Biobank recruited approximately 500,000 adults aged 40–69 years. At baseline, median age was 58 years, 54% were women, and 89% lived in England [13, 16, 17]. The cohort is not population-representative, reflecting a healthy volunteer bias [18, 19]. Approximately 157,000 (31%) UK Biobank participants have completed the Mental Health Questionnaire, with a median completion time of 14 min. Questionnaire completers were slightly younger, more often female, and healthier than the full cohort [10, 11, 14]. Our study excluded two questionnaire completers because their date of completing the questionnaire was registered as after their date of death.

### Exposures and outcomes

Our exposures were the following probable lifetime mental disorders identified by the UK Biobank Mental Health Questionnaire: (1) Depression: criteria met for depression (lifetime) on the CIDI-SF [20]; (2) Generalized anxiety disorder (sometimes abbreviated as gen. anxiety disorder in this study): criteria met for generalized anxiety disorder (lifetime) on the CIDI-SF [20]; (3) Psychotic experience: report of potential hallucination or delusion (lifetime) on questions adapted from the CIDI [21], serving as indicator of possible psychotic symptoms; (4) Bipolar disorder: criteria met for mania or hypomania (lifetime, for a week) on questions adapted from the CIDI [21]; (5) Substance use disorder: positive response to any of the following questions: “Have you been addicted to illicit or recreational drugs?” or “Have you been addicted to or dependent on prescription or over-the-counter medication?”; (6) Alcohol use disorder: positive response to the question “Have you been addicted to alcohol?”. We also generated exposures based on all possible combinations of the identified probable lifetime mental disorders.

All exposures were analyzed as binary variables (True/False). For each exposure, the reference group consisted of all Mental Health Questionnaire completers who did not meet criteria for that probable mental disorder or combination (including participants with no mental disorders and those with different mental disorders or combinations).

The UK Biobank Mental Health Questionnaire has modules that allow the identification of probable *current* mental disorders. We decided not to consider these exposures in our study. We made this decision because our exploratory data analysis showed that most combinations of *current* mental disorders identified based on the questionnaire were associated with relatively small samples and a low number of deaths, which would lead to limited analytical power.

Our outcome of interest in all analyses was time from completing the Mental Health Questionnaire to all-cause death. Mortality data were obtained via linkage with registers from the UK public National Health System and censored on September 30, 2021 [22]. Confounders in all analyses were age and sex.

### Statistical methods

Our analyses were performed in Python using the packages pandas, NumPy, statsmodels, lifelines, pycaret, and rpy2, and in R using built-in functions. Our plot was built in Microsoft Excel. Some of our code snippets were adapted from R code written by Coleman and Davis (2019) [15].

First, we performed an exploratory data analysis of all variables used in our study, including distributions, central tendency and dispersion (for numeric variables), frequencies (for categorical variables), proportions of missing values, and outliers. For that, we used a combination of univariate, bivariate, and multivariate methods, including tables and plots. Missing responses in the Mental Health Questionnaire were treated as absence of the corresponding symptom (i.e., not contributing to positive symptom scoring), and participants with missing data on core variables used to identify a specific probable mental disorder were treated as non-cases [15].

We explored combinations of probable mental disorders using an association rule mining algorithm run with the Python package pycaret. Association rule mining is a method that can be used to identify patterns of association between variables in a dataset (which corresponded to mental disorders in our study) [23]. An association rule between two sets of mental disorders X and Y can be represented as X → Y. The relevance of an association rule can be assessed using the following measurements: (I) Support (X → Y): the fraction of participants identified with both X and Y (among participants identified with at least one mental disorder) (range = [0,1]); (II) Confidence (X → Y): the fraction of participants identified with X, which were also identified with Y (range = [0,1]) (Aggarwal and Yu, 1998); (III) Lift (X → Y): the ratio of confidence (X → Y) to the support (Y) (range = [0, ∞)). The lift of an association rule can be interpreted as a measure of positive dependency (i.e., how likely participants identified with X are to be identified with Y in comparison with participants from the entire dataset) [24]. We only considered association rules with a minimum support of 0.01.

Then, we ran Cox regression models adjusted for age and sex to estimate mortality rate ratios (MRRs) associated with all possible combinations of probable mental disorders linked to at least one death. The estimated MRRs represent relative differences in mortality rates and do not directly quantify absolute mortality risk. Proportional hazards assumptions for each model were assessed using Schoenfeld residuals and showed no major violations. Minor deviations for some covariates were considered of small magnitude and did not affect conclusions. We also plotted the MRRs associated with single disorders and pairs of mental disorders linked to 10 or more deaths (which were considered as having sufficient statistical power). All MRRs are reported with 95% confidence intervals.

Additional model adjustment for socioeconomic or health-related variables was not performed because our aim was to estimate the total effect of combinations of mental disorders on mortality. These factors may act as mediators on the causal pathway between mental disorders and mortality, and adjusting for them would likely result in attenuation of the estimated effects. Consistent with this approach, a previous study by our group showed that most of the excess mortality associated with depression in the UK Biobank was explained by modifiable risk factors, including sociodemographic factors, health-related behaviors, and physical comorbidities [25].

## Results

We analyzed data from 157,314 Mental Health Questionnaire completers. Table A.1 (Supplementary material) shows the baseline characteristics of our total sample and of questionnaire completers identified with one, two, and three or more probable lifetime mental disorders. Compared with questionnaire completers identified with one mental disorder, we observed that those with combinations of mental disorders (and particularly those with three or more disorders) had an increased proportion of low income, no employment/retirement, smoking, diabetes, and previous stroke and previous myocardial infarction.

Our association rule mining algorithm was applied to a dataset including 46,683 (30%) questionnaire completers identified with at least one lifetime mental disorder. Of these participants, 13,708 (30%) had at least one additional lifetime mental disorder. Among questionnaire completers, depression was identified in 24% (*n* = 37,410), generalized anxiety disorder in 7% (11,104), psychotic experience in 5% (7,800), alcohol use disorder in 2% (3,589), bipolar disorder in 2% (2,394), and substance use disorder in 1% (1,977).

We found 602 possible association rules in our dataset, of which 40 had a support of 0.01 or more (Table A.2). The support associated with each single mental disorder was 0.80 for depression, 0.24 for generalized anxiety disorder, 0.17 for psychotic experience, 0.08 for alcohol use disorder, 0.05 for bipolar disorder, and 0.04 for substance use disorder.

Table 1 shows the top ten association rules between mental disorders according to different criteria (support, confidence, and lift). Most of the top ten association rules with highest support included depression. The specific association rules with highest support were {depression} → {gen. anxiety disorder} and vice-versa (support = 0.18) and {depression} → {psychotic experience} and vice-versa (0.08). However, all association rules with higher support had a lift lower than one (i.e., there was no evidence of positive dependency).

**Table 1:** Top association rules between probable lifetime mental disorders (*n* = 157,314, 2016–2022)

Highest support				Highest confidence				Highest lift			
Rule	S	C	L	Rule	S	C	L	Rule	S	C	L
{DD} → {GAD}	0.181	0.226	0.948	{BD, GAD} → {DD}	0.014	0.851	1.062	{AUD} → {SUD}	0.012	0.159	3.75
{GAD} → {DD}	0.181	0.76	0.948	{PE, GAD} → {DD}	0.028	0.831	1.037	{SUD} → {AUD}	0.012	0.288	3.75
{DD} → {PE}	0.078	0.097	0.583	{GAD, AUD} → {DD}	0.014	0.796	0.994	{BD, DD} → {GAD}	0.014	0.427	1.797
{PE} → {DD}	0.078	0.468	0.583	{GAD} → {DD}	0.181	0.76	0.948	{GAD} → {BD, DD}	0.014	0.06	1.797
{AUD} → {DD}	0.038	0.488	0.609	{BD} → {DD}	0.033	0.647	0.807	{BD} → {GAD, DD}	0.014	0.277	1.53
{DD} → {AUD}	0.038	0.047	0.609	{SUD} → {DD}	0.023	0.546	0.682	{GAD, DD} → {BD}	0.014	0.078	1.53
{BD} → {DD}	0.033	0.647	0.807	{AUD} → {DD}	0.038	0.488	0.609	{AUD, DD} → {GAD}	0.014	0.362	1.522
{DD} → {BD}	0.033	0.041	0.807	{PE} → {DD}	0.078	0.468	0.583	{GAD} → {AUD, DD}	0.014	0.057	1.522
{GAD} → {PE}	0.033	0.14	0.836	{BD, DD} → {GAD}	0.014	0.427	1.797	{BD} → {PE}	0.013	0.249	1.493
{PE} → {GAD}	0.033	0.199	0.836	{AUD, DD} → {GAD}	0.014	0.362	1.522	{PE} → {BD}	0.013	0.077	1.493

Probable mental disorders were identified based on the UK Biobank Mental Health Questionnaire (among questionnaire completers identified with one or more mental disorders, 46,683/157,314). Association rules are reported according to support, confidence, and lift. All selected association rules had a minimum support of 1%

S: support; C: confidence; L: lift; AUD: alcohol use disorder; BD: bipolar disorder; DD: depression; GAD: generalized anxiety disorder; PE: psychotic experience; SUD: substance use disorder

The top ten association rules with highest confidence had depression or gen. anxiety disorder on the right-hand-side. The specific association rules with highest confidence were {bipolar disorder, gen. anxiety disorder} → {depression} (confidence = 0.85), {psychotic experience, gen. anxiety disorder} → {depression} (0.83), and {gen. anxiety disorder, alcohol use disorder} → {depression} (0.80). Most association rules with higher confidence had low support and a lift lower than one.

Among the top ten association rules with highest lift, six were combinations of three disorders that included gen. anxiety disorder and depression. The association rules with highest lift were {alcohol use disorder} → {substance use disorder} and vice-versa (lift = 3.75) and {bipolar disorder, depression} → {generalized anxiety disorder} and vice-versa (1.80). Most association rules with higher lift had low support.

We studied the mortality associated with all 63 possible combinations of six probable lifetime mental disorders (Table A.3). In general, combinations of mental disorders were associated with MRR estimates of higher magnitude than single mental disorders, especially when the combinations included alcohol and substance use disorders.

**Fig. 1:**
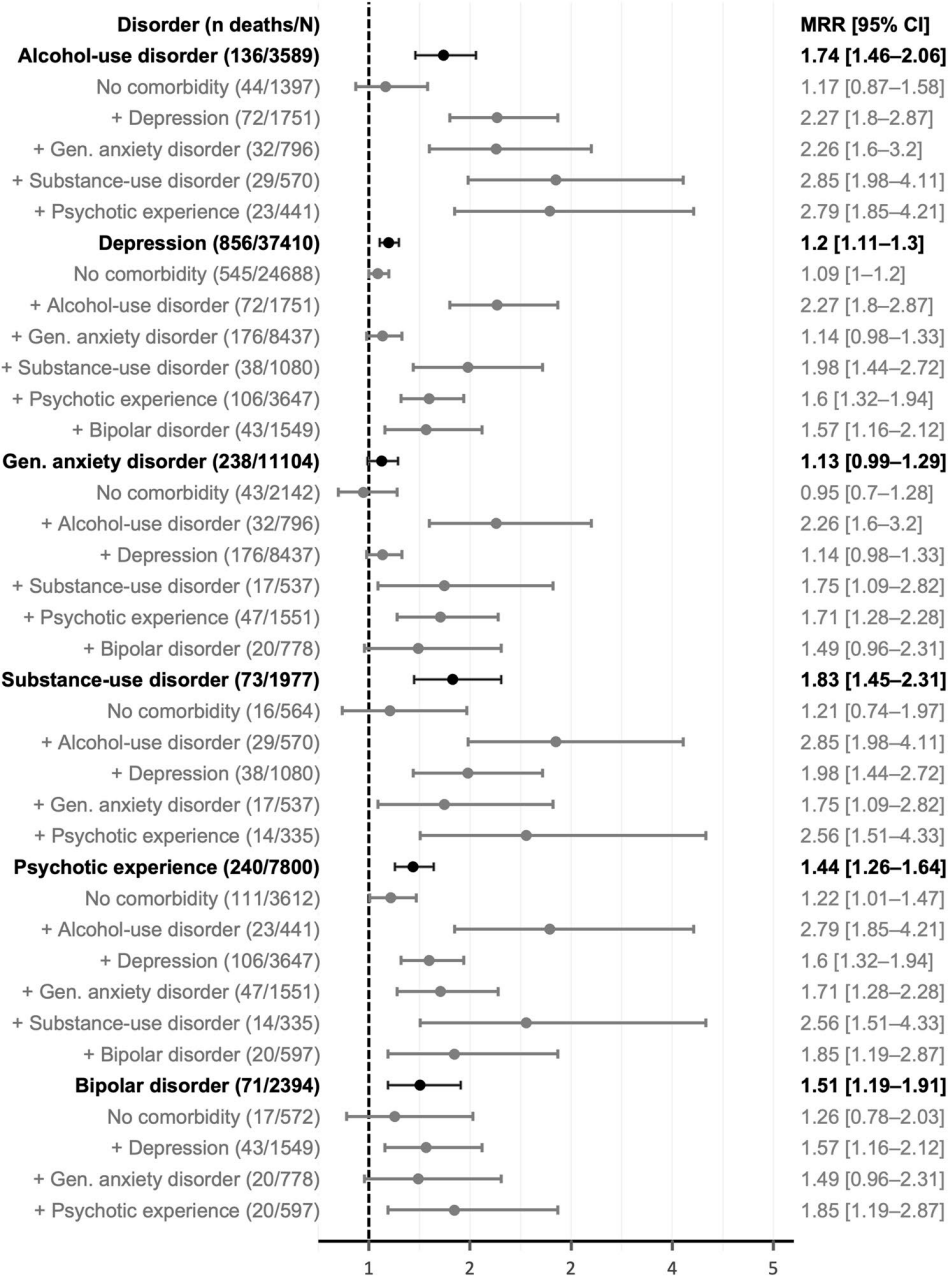
Mortality rate ratios^†^ associated with combinations of two probable lifetime mental disorders (*n* = 157,314, 2016–2022). Probable mental disorders were identified based on the UK Biobank Mental Health Questionnaire. For each disorder and combination, the figure shows the number of deaths and total participants (n deaths/N) contributing to each estimate, along with the age and sex-adjusted mortality rate ratios and 95% confidence intervals. ^†^ Plot shows only combinations of mental disorders associated with 10 deaths or more. MRR: mortality rate ratio; CI: confidence interval

When looking at pairs of mental disorders, nearly all pairs linked to 10 or more deaths were associated with increased mortality rates (Fig. 1), except generalized anxiety disorder plus depression and generalized anxiety disorder plus bipolar disorder. The pairs of mental disorders associated with the highest MRRs were alcohol use disorder plus substance use disorder (MRR = 2.85, 95% CI [1.98–4.11]), alcohol use disorder plus psychotic experience (2.79 [1.85–4.21]), and substance use disorder plus psychotic experience (2.56 [1.51–4.33]) (among those linked to 10 or more deaths).

Among all combinations of mental disorders (Table A.3), the highest MRR was associated with the combination of psychotic experience plus substance use disorder plus alcohol use disorder (MRR = 4.94 [2.57–9.5]). However, many combinations of three to six mental disorders were associated with zero to ten deaths, resulting in no MRR estimates or MRR estimates inadequately powered.

## Discussion

To our knowledge, this is the first study exploring the mortality associated with combinations of mental disorders using data from the UK Biobank Mental Health Questionnaire. The questionnaire was completed by around 160,000 UK Biobank participants and can be used to identify those with probable common lifetime mental disorders. Based on its data, we performed an association rule mining algorithm to identify patterns of association between mental disorders and estimated the mortality rate ratios associated with all possible combinations of these disorders.

Our study identified combinations of mental disorders in approximately one third of questionnaire completers identified with at least one lifetime mental disorder. As mentioned, even higher estimates (above 50%) were reported by community-based surveys, probably due to different samples and methodology [4]. The high prevalence of comorbidity in mental disorders might have several different explanations. In some cases, it can be argued that a mental disorder can trigger a subsequent mental disorder. For instance, people with depression might develop substance addiction while attempting to self-medicate negative emotions [26]. Another possible explanation for the high prevalence of comorbidity in mental disorders is that subsets of mental disorders share underlying psychopathological processes [27]. This idea is supported by studies based on factor analysis, as well as by genetic and neurodevelopmental studies. Many such studies suggested, for instance, that mood and anxiety disorders can be grouped as internalizing disorders, and substance use and conduct disorders as externalizing disorders [28–30]. Findings from these studies led to criticisms of current mental disorders classification systems for failing to capture the overlapping and dimensional nature of mental disorders [28, 29, 31]. Our findings support these concerns, as the excess mortality risk concentrated in specific combinations of mental disorders shows the limitations of single-diagnosis frameworks in capturing clinically meaningful risk.

The higher mortality associated with combinations of mental disorders shown in our study, compared with single disorders, may have several possible explanations. One is the cumulative burden of the co-occurring conditions, as individuals with multiple mental disorders may experience increased symptom load and impairment in more areas of functioning. In addition, as mentioned, they tend to present increased mental disorder severity. Indeed, in a community survey in the United States, half of the people with three or more mental disorder diagnoses were classified as severe, versus less than 10% of people with one diagnosis [5]. Mental disorder severity, in turn, seems to be associated with increased mortality in a dose-response pattern [32, 33]. For instance, a study showed that each one-point increase in depression severity measured by the 9-question Patient Health Questionnaire was associated with a 6% increase in mortality [34].

In agreement with Plana-Ripoll et al. (2020) [8], the highest MRRs in our study were observed for combinations of mental disorders that included alcohol or substance use disorders, often in combination with psychotic experience. However, these estimates were based on small numbers of deaths and should therefore be interpreted with caution. Alcohol and substance use disorders are plausibly linked to substantially increased mortality through multiple strong mechanisms, including direct toxic effects on multiple organs and an increased risk of death from external causes. It is also worth noting that these disorders were identified through direct questions about addiction or dependence, for which endorsement may reflect severe and persistent conditions that are closely associated with mortality.

In contrast, comorbid depression was generally associated with modest increases in mortality, and generalized anxiety disorder did not appear to further increase mortality risk when present in combinations. These conditions were common in mental disorder combinations in our study, suggesting that in many cases they function as transdiagnostic conditions that co-occur with other mental disorders due to shared psychopathology. Their association with mortality may be of smaller magnitude, both for single disorders and combinations, as it may reflect more indirect pathways involving the cumulative influence of multiple risk factors over time, including socioeconomic and health-related factors. Another important aspect to consider is that depression and generalized anxiety disorder were identified using symptom-based questions that tend to be sensitive and capture a broad range of severity, including milder cases that may have limited impact on mortality. It is therefore possible that increased mortality risk among individuals with these conditions is concentrated in subgroups with greater severity. Consistent with our findings, associations between depression and anxiety disorders and mortality have been heterogeneous in the literature, with meta-analyses reporting modest or variable effects depending on severity, comorbidity, and study design [3, 35].

Our findings highlight that healthcare professionals should monitor people with mental disorders for the presence of psychiatric comorbidities. Previous evidence suggests that the risk of developing additional psychiatric disorders is highest in the years following the onset of a disorder [36], highlighting a critical window for early identification and intervention. Also, from both clinical care and public health perspectives, people with combinations of mental disorders are a priority group for mortality prevention strategies. Consistent with existing frameworks endorsed by international experts, such strategies should combine the prevention and management of both mental and physical conditions, address the increased risk of death from external causes among vulnerable subgroups, and include social support interventions aimed at reducing socioeconomic disadvantage [37–40].

Our study has several limitations. These include limitations of the UK Biobank Mental Health Questionnaire, described in detail elsewhere [10, 41]. Specifically, the questionnaire relies on retrospective assessment, some not fully validated modules, and self-reported symptoms and diagnoses, which may reduce diagnostic accuracy. Thus, as mentioned, mental disorders identified through the questionnaire should be interpreted as probable conditions rather than confirmed diagnoses. Together, these limitations may have led to misclassification of mental disorders and their combinations, potentially biasing our relative mortality estimates. In addition, the questionnaire lacks information on age at onset for most disorders, which prevented its inclusion in our analyses and may have introduced immortal time bias [42]. The UK Biobank sample also shows evidence of a “healthy volunteer” selection bias, especially among Mental Health Questionnaire respondents. Studies based on population-representative samples, including individuals with greater socioeconomic disadvantage and poorer health, may generate higher estimates of relative mortality associated with mental disorders and their combinations, consistent with prior evidence [43, 44]. Another limitation is that additional adjustment for socioeconomic or health-related factors was not performed in our models because these variables may act as mediators in the pathway between mental disorders and mortality. However, residual confounding cannot be excluded. Finally, we did not formally test multiplicative interactions between mental disorders, as combinations were modeled as distinct exposure categories, allowing direct estimation of relative mortality risk. Formal interaction terms would be required to assess effect modification on the multiplicative scale, which was not the primary aim of this study.

In summary, in a large non-representative cohort of middle-aged and elderly individuals in the UK, combinations of mental disorders were commonly identified in people identified with probable lifetime mental disorders and generally associated with higher mortality rates. Future studies should continue to explore the excess mortality associated with combinations of mental disorders in different samples and contexts, along with its causes and correlates.

## Supplementary Information

Below is the link to the electronic supplementary material.

Supplementary Material 1

## Data Availability

The datasets supporting the conclusions of this article are open access and can be obtained by bona fide researchers upon application at the UK Biobank portal (https://www.ukbiobank.ac.uk).
