# Supplementary material for: Combinations of mental disorders and their association with mortality in the UK Biobank
Source: BMC Psychiatry. 2026 Feb 24;26:272. doi: 10.1186/s12888-026-07865-w (PMC13036936; doi:10.1186/s12888-026-07865-w)
Supplement: Supplementary file 1 — Supplementary Material 1 [file 12888_2026_7865_MOESM2_ESM.docx]

**Table A.1 - Baseline characteristics of participants identified with probable lifetime mental disorders and their combinations (n = 157,314, 2016-2022)**

| **Variable [Missing]** | **MHQ completers** | **No mental disorder** | **One mental disorder** | **Two mental disorders** | **≥ Three mental disorders** |
| --- | --- | --- | --- | --- | --- |
| n (%) | 157314 (100.0) | 110631 (70.3) | 32975 (21.0) | 10626 (6.8) | 3082 (2.0) |
| Age, median [Q1,Q3] | 57.0 [50.0,62.0] | 58.0 [51.0,63.0] | 55.0 [49.0,61.0] | 54.0 [48.0,60.0] | 52.0 [46.0,59.0] |
| Sex: Female, n (%) | 56.6 | 52.7 | 66.3 | 66.5 | 58 |
| Non-White ethnicity, n (%) [535] | 2.9 | 3 | 2.6 | 2.9 | 3.2 |
| Low income, n (%) [15744] | 13.8 | 12.5 | 15.7 | 18.7 | 23.8 |
| Low education, n (%) [1498] | 7 | 7.5 | 6 | 5.6 | 6.1 |
| No employment (and no retirement), n (%) [346] | 6.8 | 5.5 | 8.1 | 11.7 | 18.7 |
| Current smoker, n (%) [377] | 7.2 | 6 | 8.8 | 11.4 | 19.4 |
| Diabetes, n (%) | 3.2 | 3.1 | 3.2 | 3.6 | 4.2 |
| History of stroke, n (%) | 0.8 | 0.8 | 0.9 | 1 | 1.3 |
| History of myocardial infarction, n (%) | 1.4 | 1.4 | 1.3 | 1.4 | 1.9 |
| Deaths, n (%) | 2.5 | 2.6 | 2.4 | 2.4 | 3.3 |

Probable mental disorders were identified based on the UK Biobank Mental Health Questionnaire. Percentages are calculated excluding missing values.

MHQ: Mental Health Questionnaire

**Table A.2 - Association rules between probable lifetime mental disorders (n = 157,314, 2016-2022)**

| **Rule** | **Support** | **Confidence** | **Lift** |
| --- | --- | --- | --- |
| {SUD} → {AUD} | 0.012 | 0.288 | 3.75 |
| {AUD} → {SUD} | 0.012 | 0.159 | 3.75 |
| {BD, DD} → {GAD} | 0.014 | 0.427 | 1.797 |
| {GAD} → {BD, DD} | 0.014 | 0.06 | 1.797 |
| {BD} → {GAD, DD} | 0.014 | 0.277 | 1.53 |
| {GAD, DD} → {BD} | 0.014 | 0.078 | 1.53 |
| {AUD, DD} → {GAD} | 0.014 | 0.362 | 1.522 |
| {GAD} → {AUD, DD} | 0.014 | 0.057 | 1.522 |
| {BD} → {PE} | 0.013 | 0.249 | 1.493 |
| {PE} → {BD} | 0.013 | 0.077 | 1.493 |
| {PE, DD} → {GAD} | 0.028 | 0.353 | 1.486 |
| {GAD} → {PE, DD} | 0.028 | 0.116 | 1.486 |
| {BD} → {GAD} | 0.017 | 0.325 | 1.366 |
| {GAD} → {BD} | 0.017 | 0.07 | 1.366 |
| {SUD} → {GAD} | 0.012 | 0.272 | 1.142 |
| {GAD} → {SUD} | 0.012 | 0.048 | 1.142 |
| {BD, GAD} → {DD} | 0.014 | 0.851 | 1.062 |
| {DD} → {BD, GAD} | 0.014 | 0.018 | 1.062 |
| {PE, GAD} → {DD} | 0.028 | 0.831 | 1.037 |
| {DD} → {PE, GAD} | 0.028 | 0.034 | 1.037 |
| {GAD, AUD} → {DD} | 0.014 | 0.796 | 0.994 |
| {DD} → {GAD, AUD} | 0.014 | 0.017 | 0.994 |
| {AUD} → {GAD, DD} | 0.014 | 0.177 | 0.977 |
| {GAD, DD} → {AUD} | 0.014 | 0.075 | 0.977 |
| {GAD} → {DD} | 0.181 | 0.76 | 0.948 |
| {DD} → {GAD} | 0.181 | 0.226 | 0.948 |
| {AUD} → {GAD} | 0.017 | 0.222 | 0.932 |
| {GAD} → {AUD} | 0.017 | 0.072 | 0.932 |
| {PE} → {GAD, DD} | 0.028 | 0.165 | 0.914 |
| {GAD, DD} → {PE} | 0.028 | 0.153 | 0.914 |
| {PE} → {GAD} | 0.033 | 0.199 | 0.836 |
| {GAD} → {PE} | 0.033 | 0.14 | 0.836 |
| {BD} → {DD} | 0.033 | 0.647 | 0.807 |
| {DD} → {BD} | 0.033 | 0.041 | 0.807 |
| {SUD} → {DD} | 0.023 | 0.546 | 0.682 |
| {DD} → {SUD} | 0.023 | 0.029 | 0.682 |
| {AUD} → {DD} | 0.038 | 0.488 | 0.609 |
| {DD} → {AUD} | 0.038 | 0.047 | 0.609 |
| {PE} → {DD} | 0.078 | 0.468 | 0.583 |
| {DD} → {PE} | 0.078 | 0.097 | 0.583 |

Probable mental disorders were identified based on the UK Biobank Mental Health Questionnaire (among questionnaire completers identified with one or more mental disorders, 46,683/157,314). Association rules are reported according to support, confidence, and lift. All selected association rules had a minimum support of 1%.

AUD: alcohol use disorder; BD: bipolar disorder; DD: depressive disorder; GAD: generalized anxiety disorder; PE: psychotic experience; SUD: substance use disorder

**Table A.3 - Mortality rate ratios associated with combinations of probable lifetime mental disorders (n = 157,314, 2016-2022)**

| **DD** | **GAD** | **PE** | **BD** | **AUD** | **SUD** | **Combinations of mental disorders** | **n deaths/N** | **Mortality rate ratio (95% CI)** |
| --- | --- | --- | --- | --- | --- | --- | --- | --- |
|  |  | X |  | X | X | **PE + SUD + AUD** | 9/127 | 4.94 (2.57-9.5) |
|  | X | X |  | X | X | **PE + GAD + SUD + AUD** | 4/62 | 4.62 (1.73-12.31) |
| X | X | X |  | X | X | **PE + GAD + DD + SUD + AUD** | 3/57 | 4.12 (1.33-12.8) |
| X |  |  | X |  | X | **DD + SUD + BD** | 7/128 | 3.99 (1.9-8.38) |
| X |  | X |  | X | X | **PE + DD + SUD + AUD** | 5/103 | 3.66 (1.52-8.8) |
|  |  |  | X |  | X | **SUD + BD** | 9/157 | 3.64 (1.89-7) |
| X | X |  | X |  | X | **GAD + DD + SUD + BD** | 3/67 | 3.44 (1.11-10.68) |
| X | X |  |  | X | X | **GAD + DD + SUD + AUD** | 8/165 | 3.17 (1.58-6.34) |
|  | X |  |  | X | X | **GAD + SUD + AUD** | 10/193 | 3.09 (1.66-5.75) |
| X |  |  |  | X | X | **DD + SUD + AUD** | 17/370 | 2.92 (1.81-4.71) |
| X | X | X |  | X |  | **PE + GAD + DD + AUD** | 7/159 | 2.87 (1.37-6.03) |
|  |  |  |  | X | X | **SUD + AUD** | 29/570 | 2.85 (1.98-4.11) |
|  | X | X |  | X |  | **PE + GAD + AUD** | 8/180 | 2.84 (1.42-5.68) |
|  |  | X |  | X |  | **PE + AUD** | 23/441 | 2.79 (1.85-4.21) |
| X |  | X |  | X |  | **PE + DD + AUD** | 15/312 | 2.79 (1.68-4.63) |
|  | X |  | X |  | X | **GAD + SUD + BD** | 3/76 | 2.76 (0.89-8.58) |
|  |  | X |  |  | X | **PE + SUD** | 14/335 | 2.56 (1.51-4.33) |
| X | X |  |  | X |  | **GAD + DD + AUD** | 27/634 | 2.54 (1.74-3.72) |
| X | X |  | X | X | X | **GAD + DD + SUD + AUD + BD** | 1/33 | 2.37 (0.33-16.87) |
| X |  |  | X | X | X | **DD + SUD + AUD + BD** | 2/61 | 2.33 (0.58-9.31) |
| X |  |  |  | X |  | **DD + AUD** | 72/1751 | 2.27 (1.8-2.87) |
|  | X |  |  | X |  | **GAD + AUD** | 32/796 | 2.26 (1.6-3.2) |
|  | X | X | X |  |  | **PE + GAD + BD** | 9/254 | 2.2 (1.15-4.24) |
|  | X |  | X | X | X | **GAD + SUD + AUD + BD** | 1/37 | 2.05 (0.29-14.55) |
| X |  | X | X |  |  | **PE + DD + BD** | 15/446 | 2.04 (1.23-3.4) |
| X |  | X |  |  | X | **PE + DD + SUD** | 7/237 | 2.03 (0.97-4.27) |
| X |  |  |  |  | X | **DD + SUD** | 38/1080 | 1.98 (1.44-2.72) |
|  |  |  | X | X | X | **SUD + AUD + BD** | 2/70 | 1.96 (0.49-7.85) |
| X | X | X | X |  |  | **PE + GAD + DD + BD** | 7/227 | 1.95 (0.93-4.09) |
|  |  | X | X |  |  | **PE + BD** | 20/597 | 1.85 (1.19-2.87) |
|  |  |  |  |  | X | **SUD** | 73/1977 | 1.83 (1.45-2.31) |
|  | X | X |  |  | X | **PE + GAD + SUD** | 4/153 | 1.83 (0.68-4.87) |
| X | X |  |  |  | X | **GAD + DD + SUD** | 13/446 | 1.77 (1.03-3.06) |
|  | X |  |  |  | X | **GAD + SUD** | 17/537 | 1.75 (1.09-2.82) |
|  |  |  |  | X |  | **AUD** | 136/3589 | 1.74 (1.46-2.06) |
| X | X | X |  |  | X | **PE + GAD + DD + SUD** | 3/133 | 1.73 (0.56-5.37) |
|  | X | X |  |  |  | **PE + GAD** | 47/1551 | 1.71 (1.28-2.28) |
| X | X | X |  |  |  | **PE + GAD + DD** | 37/1289 | 1.69 (1.22-2.33) |
| X |  | X |  |  |  | **PE + DD** | 106/3647 | 1.6 (1.32-1.94) |
| X |  |  | X |  |  | **DD + BD** | 43/1549 | 1.57 (1.16-2.12) |
|  |  |  | X |  |  | **BD** | 71/2394 | 1.51 (1.19-1.91) |
|  | X |  | X |  |  | **GAD + BD** | 20/778 | 1.49 (0.96-2.31) |
| X |  |  | X | X |  | **DD + AUD + BD** | 4/160 | 1.48 (0.56-3.95) |
|  |  | X |  |  |  | **PE** | 240/7800 | 1.44 (1.26-1.64) |
| X | X |  | X |  |  | **GAD + DD + BD** | 15/662 | 1.35 (0.81-2.24) |
|  |  |  | X | X |  | **AUD + BD** | 5/206 | 1.34 (0.56-3.22) |
|  |  |  | X |  |  | **BD (no comorbidity)** | 17/572 | 1.26 (0.78-2.03) |
|  |  | X |  |  |  | **PE (no comorbidity)** | 111/3612 | 1.22 (1.01-1.47) |
|  |  |  |  |  | X | **SUD (no comorbidity)** | 16/564 | 1.21 (0.74-1.97) |
| X |  |  |  |  |  | **DD** | 856/37410 | 1.2 (1.11-1.3) |
|  |  |  |  | X |  | **AUD (no comorbidity)** | 44/1397 | 1.17 (0.87-1.58) |
| X | X |  |  |  |  | **GAD + DD** | 176/8437 | 1.14 (0.98-1.33) |
|  | X |  |  |  |  | **GAD** | 238/11104 | 1.13 (0.99-1.29) |
| X |  | X | X | X |  | **PE + DD + AUD + BD** | 1/57 | 1.11 (0.16-7.88) |
| X |  |  |  |  |  | **DD (no comorbidity)** | 545/24688 | 1.09 (1-1.2) |
|  | X |  |  |  |  | **GAD (no comorbidity)** | 43/2142 | 0.95 (0.7-1.28) |
|  |  | X | X | X |  | **PE + AUD + BD** | 1/69 | 0.89 (0.12-6.3) |
| X | X |  | X | X |  | **GAD + DD + AUD + BD** | 1/83 | 0.75 (0.11-5.33) |
|  | X |  | X | X |  | **GAD + AUD + BD** | 1/99 | 0.63 (0.09-4.48) |
| X |  | X | X | X | X | **PE + DD + SUD + AUD + BD** | - | - |
| X |  | X | X |  | X | **PE + DD + SUD + BD** | - | - |
|  | X | X | X | X |  | **PE + GAD + AUD + BD** | - | - |
| X | X | X | X | X |  | **PE + GAD + DD + AUD + BD** | - | - |
| X | X | X | X | X | X | **PE + GAD + DD + SUD + AUD + BD** | - | - |
| X | X | X | X |  | X | **PE + GAD + DD + SUD + BD** | - | - |
|  | X | X | X | X | X | **PE + GAD + SUD + AUD + BD** | - | - |
|  | X | X | X |  | X | **PE + GAD + SUD + BD** | - | - |
|  |  | X | X | X | X | **PE + SUD + AUD + BD** | - | - |
|  |  | X | X |  | X | **PE + SUD + BD** | - | - |

Probable mental disorders were identified based on the UK Biobank Mental Health Questionnaire. For each disorder and combination, the table shows the number of deaths and total participants (n deaths/N) contributing to each estimate, along with the age and sex-adjusted mortality rate ratios and 95% confidence intervals.

AUD: alcohol use disorder; BD: bipolar disorder; DD: depressive disorder; GAD: generalized anxiety disorder; PE: psychotic experience; SUD: substance use disorder; CI: confidence interval
